# Biofilm formation by enteric pathogens and its role in plant colonization and persistence

**DOI:** 10.1111/1751-7915.12186

**Published:** 2014-10-29

**Authors:** Sima Yaron, Ute Römling

**Affiliations:** 1 Faculty of Biotechnology and Food Engineering, Technion – Israel Institute of TechnologyHaifa, 32000, Israel; 2 Department of Microbiology, Tumor and Cell Biology, Karolinska InstitutetStockholm, Sweden

## Abstract

The significant increase in foodborne outbreaks caused by contaminated fresh produce, such as alfalfa sprouts, lettuce, melons, tomatoes and spinach, during the last 30 years stimulated investigation of the mechanisms of persistence of human pathogens on plants. Emerging evidence suggests that *Salmonella enterica* and *Escherichia coli*, which cause the vast majority of fresh produce outbreaks, are able to adhere to and to form biofilms on plants leading to persistence and resistance to disinfection treatments, which subsequently can cause human infections and major outbreaks. In this review, we present the current knowledge about host, bacterial and environmental factors that affect the attachment to plant tissue and the process of biofilm formation by *S. enterica* and *E. coli*, and discuss how biofilm formation assists in persistence of pathogens on the plants. Mechanisms used by *S. enterica* and *E. coli* to adhere and persist on abiotic surfaces and mammalian cells are partially similar and also used by plant pathogens and symbionts. For example, amyloid curli fimbriae, part of the extracellular matrix of biofilms, frequently contribute to adherence and are upregulated upon adherence and colonization of plant material. Also the major exopolysaccharide of the biofilm matrix, cellulose, is an adherence factor not only of *S. enterica* and *E. coli*, but also of plant symbionts and pathogens. Plants, on the other hand, respond to colonization by enteric pathogens with a variety of defence mechanisms, some of which can effectively inhibit biofilm formation. Consequently, plant compounds might be investigated for promising novel antibiofilm strategies.

## Introduction

The number of outbreaks of foodborne illness arising from the consumption of fresh and fresh-cut produce increased dramatically two decades ago and has, since then, continued to be high in both, absolute numbers of outbreaks and relative numbers compared with other foodborne outbreaks with an identified source (Anonymous, 2008; Olaimat and Holley, 2012; CDC, 2014). Microorganisms that have been frequently associated with illness related to consumption of fresh produce include bacteria as diverse as *Salmonella enterica* serovars, *Escherichia coli* pathovars, *Listeria monocytogenes*, *Bacillus cereus*, *Vibrio cholerae*, *Shigella* spp., *Campylobacter* spp., *Yersinia enterocolitica*, *Aeromonas hydrophila* and *Clostridium* spp.; viruses such as norovirus and hepatitis A; and protozoa such as *Cyclospora cayetanensis* and *Cryptosporidium parvum*. Specific types of fresh foods that have been identified as common sources in produce-associated outbreaks include sprouts, green leaves like lettuce and spinach, and fruits and vegetables like melons and tomatoes (Doyle and Erickson, 2008; Yaron, 2014). *Salmonella enterica* and *E. coli* are the two major species that cause large outbreaks of foodborne illness associated with fresh produce. *Salmonella enterica* is more frequent in outbreaks caused by fruits, seeds and sprouts, while *E. coli* O157:H7 is more frequent in leafy greens (Brandl, 2006).

Since fruits, vegetables and leafy greens are typically consumed without thermal treatment, outbreaks originating from such food sources usually affect a large number of individuals. An example is the recent *E. coli* O104:H4 outbreak in North Germany in 2011. A newly emerged *E. coli* O104:H4 strain caused the highest frequency of haemolytic uremic syndrome and death ever recorded in a single *E. coli* outbreak. Seeds of fenugreek imported from Egypt were likely the source of the outbreak (Mariani-Kurkdjian and Bingen, 2012).

Another problem associated with enteric pathogens linked to fresh produce is relating to the fact that washing of produce with chlorine or other antimicrobial solutions fails to significantly reduce the attached pathogens (Beuchat, 1997; Gandhi *et al*., 2001; Kondo *et al*., 2006). Most of the available literature regarding the use of chemicals for washing has concluded that each treatment reduces the pathogens associated with the produce by no more than 3 logs, and usually less than 1 log (Beuchat *et al*., 2004; Gonzalez *et al*., 2004; Allende *et al*., 2007; Shirron *et al*., 2009). Moreover, recent evidence has shown that enteric pathogens are less susceptible to common sanitizing agents like chlorine than the indigenous microorganisms, suggesting that after sanitizing, remaining pathogens can survive and regrow on the wet products with less competition (Shirron *et al*., 2009).

Plants were commonly considered not to support the persistence and colonization of enteric pathogens. Until recently, the conventional view was that bacterial enteric pathogens such as *E. coli* O157:H7 and *S. enterica* survive poorly in the harsh environment encountered on plant surfaces. The raise in produce-borne outbreaks during the last decades has evoked intensive surveys of fresh produce products. These studies indicate that contamination of fresh produce with foodborne pathogens might occur more frequently than previously thought. For example, surveillance studies to determine the incidence of *S*. *enterica* serovars on farm and retail products have shown that the prevalence of *S*. *enterica* ranges from 0% to as high as 35.7% of the sampled foods (Doyle and Erickson, 2008). However, it seems that routine testing of fresh produce using standard recovery methods may fail in recognition of contaminations, because in cases of low abundance of the pathogens, such methods may not be sensitive enough to detect the presence of the pathogens, resulting in underestimation of the contamination frequency (Kisluk *et al*., 2012). Furthermore, it was reported that pathogens form aggregates or biofilms (Brandl and Mandrell, 2002), or alternatively can evolve into a viable but non-cultivable (VBNC) state on plants (Dinu and Bach, 2011). The limited ability to enumerate aggregated bacteria or to detect low levels of the pathogens, and the possibility of induction of VBNC cells in plants are a source of concern, since the infective dose in several large outbreaks was considered to be as low as a few cells (Lehmacher *et al*., 1995; Collignon and Korsten, 2010; Kisluk *et al*., 2012).

Recent analyses of outbreaks associated with identified contaminated sources showed that contamination of at least 20% of the products occurred on the farm, while the rest of the outbreaks was associated with improper handling of produce after leaving the farm (Yaron, 2014). Contamination of fresh produce is aided as enteric pathogens are able to survive on the produce in the field or post-harvest for long periods of time although their overall populations most often decline after inoculation (Brandl and Mandrell, 2002; Brandl, 2006; Kisluk and Yaron, 2012). For example, *S*. Typhimurium inoculated on parsley or basil survived for at least 100 days on the leaves (Kisluk and Yaron, 2012; Kisluk *et al*., 2013), *E. coli* O157:H7 survived on parsley 177 days (Islam *et al*., 2004) and *E. coli* O104:H4 survived even better than *E. coli* O157:H7 on spinach, basil and lettuce (Markland *et al*., 2012). In all of these examples, the bacteria survived without causing disease symptoms *in planta*. Although these microorganisms are considered to be adapted to colonize warm- and cold-blooded animals, enteric bacteria are usually exposed to a new host via contaminated foods or water, and excreted back to the environment through the animal feces. As these pathogens persist for a certain time in the environment, plants may serve as potential vehicles for their transfer from the environment to a new host (Ochman and Groisman, 1995). Consequently, enteric bacteria not only survive, but also replicate on the plants until the plant is consumed by a new potential host. Thus, it is reasonable to propose intimate interactions between the bacteria and the plant (Shirron and Yaron, 2011), interactions that recently have begun to be scrutinized (Hernandez-Reyes and Schikora, 2013).

One of the most fascinating strategies to gain fitness against the challenging conditions on or in the plant is the formation of biofilms. Microbial biofilms can be formed on leaves, on root surfaces and also within intercellular spaces of plant tissues. As a benefit, biofilm formation protects attached bacteria from desiccation, UV radiation and other environmental stresses, as well as from the plant immune response and from antimicrobial compounds produced by the plant or by indigenous microorganisms. The ability to form biofilms also provides enhanced protection against chemicals used for disinfection during processing of the food (Scher *et al*., 2005; Lapidot *et al*., 2006). This review will present the factors affecting the attachment to and the process of biofilm formation on plant tissue by foodborne pathogens, and will discuss the topic of how biofilm formation assists in persistence of pathogens on the plants. Although a variety of pathogens have been implicated in outbreaks arising from produce, this review will focus primarily on *S. enterica* and *E. coli* because of the high frequency of outbreaks associated with these pathogens and the relative depth to which these foodborne pathogens have been studied in relation to biofilm formation on plants.

## The plant environment and bacterial survival strategies

In order to understand the fate of enteric pathogens on plants, it is important to be familiar with the conditions the bacteria face in the plant environment. Depending on the route of transmission (water, manure, improper handling and other measures), bacteria may be located in the rhizosphere or the phyllosphere. The root zone in the soil is relatively rich in nutrients, thus supporting the persistence of 10^6^ to 10^9^ bacteria per gram of roots (Hallmann *et al*., 2001). The rhizosphere contains root exudates including compounds released as a consequence of root cell metabolism or after lysis of plant cells. A major compound of root secretions is mucilage composed of hydrated polysaccharides, organic acids, vitamins and amino acids which are excellent substrates for microbial growth. Mucilage binds water and thus helps to form a well-hydrated environment for the roots and rhizosphere microorganisms. Some bacteria that colonize the root surface are able to infect the vascular parenchyma followed by invasion into the xylem vessels and transfer to the upper parts of the plants (Kutter *et al*., 2006; Klerks *et al*., 2007).

Unlike the rhizosphere, nutrients are scarce on the foliage surface. The few plant-derived nutrients on leaves probably originate from mesophyll and epidermal cell exudates leaking onto the surface as well as from wounds and broken trichomes. The distribution of these nutrients is highly heterogeneous. Moreover, the phyllosphere is subjected to large and rapid fluctuations in temperature, solar radiation and water availability, and therefore typically supports fewer than 10^3^ to 10^7^ bacteria per gram leaf (Hallmann *et al*., 2001). These environmental conditions differ significantly from the comparatively weak and buffered fluctuations of abiotic conditions prevailing in the rhizosphere or the rich and relatively stable environment in the intestine of animals. Foliar bacteria may follow two major strategies for their growth and survival on the plant surface: A tolerance strategy that requires the ability to resist exposure to environmental stresses on leaf surfaces or an avoidance strategy by which the bacteria seek sites that are protected from those stresses (Beattie and Lindow, 1999). Based on these strategies, a general model of leaf colonization was developed. According to this model, the bacteria that arrive on the leaf surface are randomly distributed. Some bacteria enter into the leaf via openings such as stomata, and those that stay on the surface modify their local environment. The bacteria adhere to the surface, start to multiple and form aggregates or microcolonies, which may be further developed into biofilms. Some bacteria continue to invade into internal spaces, in which they modify the habitat.

Knowledge about the behaviour of human enteric pathogens on plants has just begun to accumulate. It is however emerging that those ‘non-professional’ plant-interacting organisms use similar mechanisms with plants as described above (Brandl, 2006). Using a similar strategy for survival, the main difference between plant and human enteric pathogens is that no significant multiplication on leaves surfaces of mature plants is observed for enteric pathogens, though growth was observed under specific conditions such as on cut products (Pan and Schaffner, 2010) or during germination of sprouts (Gandhi *et al*., 2001). In addition, in most cases, enteric pathogens survive on or in the leaf without significant changes of the habitat, and thus, without visible symptoms. These bacteria rarely modify the plant structure, but tend to aggregate or to form biofilms as will be discussed in next sections.

## Bacterial biofilms

Biofilms are complex communities of microorganisms in which cells are attached to a surface and to each other, and are embedded in a self-produced matrix of extracellular polymeric substances (EPS) (Costerton *et al*., 1999). The major component of biofilms is actually water (up to 97%) and bacterial cells make up to 35% of the dry weight. Apart of live and dead bacteria, a variety of secreted compounds such as polysaccharides, proteins, lipopolysaccharides (LPS), DNA and lipids contribute to the dry weight of the biofilm in addition to minerals and other components from lysed or dead cells or from the environment (like host components) that jointly form the biofilm matrix (Costerton *et al*., 1999).

Development of bacterial biofilms on surfaces typically involves several stages, which are likely to occur also on the surface of plants. The initial stages of biofilm formation depend on bacterial motility which enables the free-swimming bacteria to reach a suitable surface (Blair *et al*., 2008). Consequently, the flagella act as motility organelles that assist in arrival to favourable habitats and can be adhesion factors that promote attachment to the surface. Stringent regulation of flagella rotation and functionality is subsequently required for optimal biofilm formation. For example, in *Bacillus subtilis*, disengaging the flagellum from the rotor facilitated the transition to the biofilm state (Blair *et al*., 2008). Next, the bacteria adhere to the surface, irreversibly attach to it, form microcolonies and secrete EPS that are required for the interactions of the cells with the surface, with other cells and with alternative matrix components to develop the complex architecture of the biofilm. Proteins in the biofilm matrix carry out primarily both structural and physiological functions. Exopolysaccharides confer mechanical stability and have a role in water retention and nutrient availability. In late stages of biofilm development, the microcolonies develop into mature biofilms with complex three-dimensional structures. Bacteria may actively or passively detach from the biofilm, and dispersed individual cells or clumps may spread into a new environment. Environmental signals, quorum sensing and cyclic dimeric guanosine monophosphate (di-GMP) secondary messenger signalling are major components to regulate the different stages of the biofilm developmental process (Blair *et al*., 2008; Ahmad *et al*., 2013). Consequently, mature biofilms are dynamic heterogenic environments.

Cells in the biofilm are more resistant to chemicals, stress conditions and components of the host immune system (Costerton *et al*., 1999), and thus it was suggested that the formation of biofilms by bacterial cells on plant surfaces is a survival strategy to withstand the harsh conditions in this environment. Several mechanisms contribute to the enhanced resistance of biofilm-associated cells, which also depend on the property of the antimicrobial compound and the genetic potential of the bacterial strain (reviewed in del Pozo and Patel, 2007). For example, EPS can provide a physical barrier against the diffusion of antimicrobial agents and compounds of the defence response and offers protection against environmental stress factors such as UV radiation, osmotic stresses and desiccation.

Like other species, the ecological success of enteric pathogens such as *S. enterica* and *E. coli* in a variety of hosts, including plants, and in different niches in the environment is in part due to their ability to grow in biofilm (Costerton *et al*., 1995; Davey and O'Toole, 2000). These species form biofilms on abiotic surfaces such as stainless steel and glass (Joseph *et al*., 2001; Zogaj *et al*., 2001; Kim and Wei, 2007; Schlisselberg and Yaron, 2013), on surfaces in the host such as the epithelial cell layer and gallstones (Prouty and Gunn, 2003; Esteves *et al*., 2005), and on plant surfaces (Mahon *et al*., 1997; Campbell *et al*., 2001; Franz *et al*., 2007). Additional biofilms are pellicles at the air–liquid interface (Anriany *et al*., 2001; Scher *et al*., 2005; 2007), biofilms colonizing cancer tissue, food stuff, equipment in the food industry and biofilms occurring under many more circumstances (Thomas and McMeekin, 1981; Craven and Williams, 1998; Prouty *et al*., 2002; Winfield and Groisman, 2003; Chia *et al*., 2009; Vestby *et al*., 2009; Crull *et al*., 2011).

## Reversible and irreversible attachment of native bacteria and enteric pathogens to plant tissue

As mentioned above, attachment is an initial step crucial for biofilm formation on the plant surface. Analysis of attachment of plant pathogens and symbionts such as *Rhizobium* and *Agrobacterium* to the root or leaf surfaces showed a biphasic process that occurs after bacterial contact with plant surfaces. In the first few seconds, the initial adhesion is characterized by a weak, reversible and unspecific binding that usually depends on hydrophobic and electrostatic interactions. In the second phase of binding, a strong irreversible attachment might occur (Dunne, 2002). This form of attachment has also been called ‘firm’ attachment, since removal of the attached bacteria cannot be readily achieved. In many symbionts, the second attachment step involves bacterial cellulose fibres (Laus *et al*., 2005).

Studies of the attachment of human enteric pathogens indicate that they can rapidly adhere to a variety of plant tissues (leaves, fruits, roots) of growing or harvested plants using a similar scheme of attachment. Attachment is irreversible, since bacteria are not removed by washing. Table 1 lists studies on the attachment of *E. coli* and *S. enterica* serovars to different plants types and plant tissues. Adhesion studies conducted for more than 4 h were excluded, because after a long time, attached bacteria may die, or, alternatively, particularly in sprouts or cut plant tissues, can grow, so it is impossible to discriminate attachment, from other processes such as survival and growth. Exemplified in Table 1, ubiquitously a firm attachment was obtained within few seconds to less than few hours as depending on the detection time. More than qualitative comparisons are however not applicable due to major differences in the experimental set-up, including preparation of the inoculum, concentration of the bacteria in the binding assay, type of liquid (water, saline, buffer, etc.), temperature of the assay, methods used to recover the attached bacteria and different reports of result output parameters.

**Table 1:** Adhesion and attachment of *Salmonella* and *E. coli* to plants tissues

Pathogen	Plant/part	Time	No. attached	Comments	Reference
*E. coli* O157:H7	Cut green pepper	2 h	6.6–7.3 log cfu g^−1^	Injured fruits also investigated	(Han *et al*., 2000)
*E. coli* O157:H7 *Salmonella* serovars	Alfalfa sprouts	4 h	2.8–3 log cfu per sprout 3.0–3.3 log cfu per sprout		(Barak *et al*., 2002)
*E. coli* O157:H7	Arugula leaves	1 h	2 log cfu cm^−2^	Mutants investigated	(Shaw *et al*., 2008)
*E. coli* O157:H7 *Salmonella* serovars	Peach fruits	60 s	1.6 log cfu cm^−2^ 4.1 log cfu cm^−2^		(Collignon and Korsten, 2010)
*E. coli* O157:H7 *Salmonella* serovars	Plum fruits	30 s	0.6 log cfu cm^−2^ 3.2 log cfu cm^−2^		(Collignon and Korsten, 2010)
*E. coli* O157:H7	Spinach leaves	< 1 h	2.7–2.9 log cfu per leaf	Mutants investigated	(Macarisin *et al*., 2012)
*E. coli* O157:H7 *E. coli* K-12	Lettuce leaves	0.25–2 h	1–2.5 log cfu cm^−2^	Mutants investigated	(Fink *et al*., 2012)
*S.* Stanley	Cantaloupes	10 min	3.8 log cfu cm^−2^		(Ukuku and Sapers, 2001)
*S.* Chester	Cut green pepper	30 s	5.85 log cfu per disc (56 mm^2^)		(Liao and Cooke, 2001)
*S.* Montevideo	Tomatoes	1.5 h	4–5.4 log cfu per fruit	After few sec the attachment is fourfold lower. Type of product, temperature and relative humidity affect attachment	(Iturriaga *et al*., 2003)
*S.* Newport	Alfalfa sprouts	1 h	700–800 cfu per sprout	Mutants investigated	(Barak *et al*., 2005)
*S.* Typhimurium	Parsley leaves	1 h	7.4 log cfu g^−1^	Mutants investigated	(Lapidot *et al*., 2006)
*S.* Enteritidis	Alfalfa sprouts	1 h	2.3 log cfu per sprout	Mutants investigated	(Barak *et al*., 2007)
*S.* Typhimurium and Senftenberg	Basil, lettuce, rocket and spinach leaves	1 h	200–250 cfu mm^−2^	Mutants investigated. Attachment is affected by temperature in Senftenberg.	(Berger *et al*., 2009)
*S.* Typhimurium	Parsley leaves Cucumber fruits	30 min	6.1 log cfu g^−1^ 5.1 log cfu g^−1^		(Shirron *et al*., 2009)
*S.* serovars Negev, Newport, Tennessee, Thompson, Braenderup	Intact and cut lettuce (Romaine, Iceberg) and cabbage	1–4 h	2–4 log cfu cm^−2^	Attached to Romaine lettuce at higher numbers than those attached to Iceberg lettuce or cabbage. Differences between serovars. Attached preferentially to cut surface of all produce. Attachment increased with time.	(Patel and Sharma, 2010)
*S.* Typhimurium	Cut Romaine lettuce leaves	2 h	6.4–7.7 log cfu g^−1^	Levels depend on the leaf region and age. Maximal attachment is near the petiole or on older leaves.	(Kroupitski *et al*., 2011)
*S.* Typhimurium, Senftenberg and Thompson	Tomato fruits	1 h	4.9–5.1 log cfu mm^−2^	Mutants investigated	(Shaw *et al*., 2011)
*S.* Typhimurium and Saintpaul	Intact spinach leaves Grape tomatoes	10 min	6–7 log cfu per 3 leaves 5–6 log cfu per 3 fruits	Mutants investigated	(Salazar *et al*., 2013)

Besides the bacterial inoculum and exposure time, both host plants and bacterial properties influence the efficacy by which the enteric pathogens attach to plants. Attachment to basil, lettuce or spinach leaves differed among *S. enterica* serovars, as *S*. Senftenberg and *S*. Typhimurium showed higher attachment compared with *S*. Agona or *S*. Arizonae. Interestingly, the *S*. Senftenberg strain with highest adhesion capability to basil was a clinical isolate from a basil-derived outbreak in the UK in 2007 (Berger *et al*., 2009). Microscopic observations of three *Salmonella* serovars attached to tomato fruits show that although all investigated serovars were attached to tomatoes with similar efficiencies, serovars Senftenberg and Typhimurium adhered to the fruits in an aggregative pattern, while serovars Thompson adhered in a diffuse pattern (Shaw *et al*., 2011). Enteric pathogens such as *E. coli*, *Salmonella* and *Listeria* adhered more effectively to the peach fruit than plum surfaces attributed to the increased surface area of the peach fruits due to the presence of trichomes (Collignon and Korsten, 2010). Also, in line with epidemiological data, the affinity of *Salmonella* serovars to lettuce was significantly twofold to threefold higher than to cabbage (Patel and Sharma, 2010). Lettuce is very often associated with foodborne outbreaks, whereas outbreaks associated with cabbage are rare.

Environmental factors affect the attachment as well. The adhesion of pathogens in wash water to fresh cucumber surfaces depends on temperature, and is less extensive at lower temperatures. The effect of dewaxing of fruits on adhesion depends on the bacteria. While adhesion of *Listeria* to dewaxed fruits was higher than to waxed fruits, the opposite was reported for *S*. Typhimurium and *Staphylococcus aureus* (Reina *et al*., 2002).

## Factors that play a role in attachment of *S. enterica* and *E. coli* to plants

### Properties of plant surfaces

Most aerial plant surfaces are covered in cuticle, a hydrophobic material composed primarily of fatty acids, waxes and polysaccharides. The cuticle favours attachment of hydrophobic molecules. However, breaks in the cuticle may expose hydrophilic structures (Patel and Sharma, 2010). In this case and on the root surface, the bacteria are exposed to the plant cells, which are generally covered with glycoproteins and polysaccharides such as cellulose and pectins. Many of these molecules are hydrophilic and in some cases have negative charge (Torres *et al*., 2005). Plant surface charge correlates with the strength of attachment (Ukuku and Fett, 2002; 2006), but the exact receptors or binding sites, if existent, have not been identified. Investigation of attachment of *S*. Typhimurium to sliced potatoes indicated that the bacteria attach to cell wall junctions. In particular, the bacteria appeared to attach to the pectin layer at the junctions, indicating that pectin may be the bacterial attachment site (Saggers *et al*., 2008). In contrary, Tan and colleagues have shown that *Salmonella* attached in lower numbers to plant cell wall components when pectin was part of the composite, supporting that pectin is unfavourable for the bacterial attachment compared with cellulose (Neff *et al*., 1987; Tan *et al*., 2013).

Topography and architecture of the surface of the plant are also important factors in microbial adhesion. Roughness is important not only for adherence but also for survival on the plant tissue, as demonstrated for *E. coli* O157:H7 adhesion on leaves of different spinach cultivars (Macarisin *et al*., 2013). The surface roughness of the plant organs such as leaves depends on the nature of the plant and on the age of the leaves. Indeed, the affinity of *Salmonella* to artificially contaminated old lettuce leaves was higher compared with young leaves. Moreover, higher numbers of *S*. Typhimurium were localized close to the petiole, and the bacteria displayed higher affinity towards the abaxial side compared with the adaxial side of the leaves (Kroupitski *et al*., 2011). Fissures in the cantaloupe netting provided attachment sites for cells of *Salmonella* which aid bacterial survival when in contact with aqueous sanitizers (Annous *et al*., 2004; 2005).

The plant microflora is not homogenously distributed on the leaf surface, rather bacterial cells have been shown to attach and colonize at specific sites in and on leaf surfaces, including the base of trichomes, at stomata, epidermal cell wall junctions, as well as in grooves along veins and depressions or beneath in the cuticle (Beattie and Lindow, 1999). These habitats apparently constitute stress-protected, rich-in-water and rich-in-nutrients sites. Plant appendages such as secretory cavities or ducts may release plant metabolites. Glandular trichomes are epidermal protuberances which serve as sites of secretion and accumulation of different compounds such as Ca, Na, Mn and Pb ions, defensive proteins and secondary metabolites such as essential oils, monoterpenoids and phenylpropanoids. Larger numbers of bacteria can also be found on the lower than upper leaf surface, possibly due to lower radiation exposure, or because of higher density of stomata or trichomes and a thinner cuticular layer (Karamanoli *et al*., 2012). Consequently, bacteria attached on the lower leaf surface find better conditions for survival and growth, which increase the probability of their survival compared with bacteria attached to other parts of the leaf.

Evidence indicates that human enteric pathogens demonstrate similar behaviour on leaves, with a few differences. *Salmonella enterica* serovar Thompson was shown to attach around stomata of spinach leaves and in cell margins, similar to where native bacteria are detected (Warner *et al*., 2008). Use of confocal microscopy to visualize cells of *E. coli* attached at stomata and trichomes of cut lettuce plants concluded that attachment sites for *E. coli* are similar to those reported for plant pathogens (Seo and Frank, 1999). The stomata provide protective niches for the bacteria, and also can serve as a source of nutrients. Golberg and colleagues confirmed that *Salmonella* cells prefer this niche by showing that the bacteria are mostly located near and within the stomata of lettuce leaves. However, the ability of *Salmonella* to colonize the surface around the stomata was observed only with certain serovars on specific plants (Golberg *et al*., 2011). On the other hand, while *E. coli* better attached to cut surfaces of lettuce, *Pseudomonas fluorescens* preferentially attached to the intact areas, and *S*. Typhimurium attached to both, cut and intact surfaces in a similar manner (Takeuchi *et al*., 2000). Whether the ability of enteric pathogens to localize to similar adhesion sites on the leaves like plant pathogens or the natural microflora contributes to long-term survival in the plant environment is an issue that should be addressed.

Enteric bacteria penetrating into the soil through water, fertilizers or directly exposed to the roots during hydroponic growth, are able to attach to the rhizosphere of the plant host. Following attachment, these bacteria can invade or move to the upper parts of the plant (Lapidot and Yaron, 2009). In the case of attachment to the root surface, in contrast to leaves and fruits, more significant differences were observed between the location of the natural microflora and enteric pathogens. The natural plant microflora and plant pathogens tend to attach to the epidermis and to the root hairs formed by trichomes. Plant pathogens bind rapidly and particularly well to cut ends of roots and wound sites and bind poorly to the root tips (Matthysse and Kijne, 1998). In contrast, *E. coli* strains prefer to attach to the root tips of alfalfa sprouts, but attach to the roots very slowly. Further, not all investigated *E. coli* strains are able to bind to the root hairs (Jeter and Matthysse, 2005).

### Bacterial properties

It is mostly believed that attachment of *Salmonella* and *E. coli* is an active process, but not all observations support this assumption. Only viable *Salmonella* cells were able to attach to vegetable tissues such as slices of potatoes (Saggers *et al*., 2008). On the other hand, similar levels of attachment to lettuce were observed with live *E. coli* O157:H7, killed *E. coli* O157:H7 and fluorescent polystyrene microspheres (Solomon and Matthews, 2006). The difference was attributed to the method used for bacterial inactivation. *Escherichia coli* cells were inactivated with glutaraldehyde, which is known to alter the adhesive properties of the bacterial envelope, while *Salmonella* cells were inactivated by different methods including formalin, ethanol, kanamycin and thermal treatment (Saggers *et al*., 2008).

A number of authors investigated the role of cell surface charge, presence of divalent cations, hydrophobicity and capsule production in passive or active attachment of *E. coli* to lettuce tissue (Hassan and Frank, 2003; 2004; Boyer *et al*., 2007). Collectively, these studies have shown very little correlation between the presence of cell surface appendages, charge or hydrophobicity and the ability of the bacteria to attach to lettuce tissue. Subsequently, treatment with the hydrophobic surfactant Span85 detached only 80% of attached *E. coli* O157:H7 from intact lettuce leaves, and this surfactant was ineffective in detaching the pathogen from cut edges, indicating that the nature of surface is heterogeneous (Hassan and Frank, 2003). Alternatively, for *Salmonella*, a linear correlation was reported between bacterial cell surface hydrophobicity and the strength of attachment to melon fruits (Ukuku and Fett, 2002; 2006).

On the molecular level, studies investigating the role of specific bacterial factors in adhesion to plants have shown contradictory results, and until now, very few genetic elements have been definitively identified as essential for attachment or survival of human pathogens on leaves, roots, fruits or sprouts. The specific bacterial factors that contribute to attachment to plant tissue were identified by different experimental approaches like differential expression analysis upon contact with plants or plants extracts and assessment of the ability of mutants and overexpression strains to attach to plant tissues. Table 2 presents major genes frequently identified and investigated. Interestingly, many genes that have a role in adhesion to plants tissues have also been identified as attachment or virulence factors of *E. coli* and *S.* Typhimurium when infecting animals. This phenomenon occurs despite of the fact that many studies not only focused on genes with known function in the host, but also screened for functional genes using whole genome transcription analysis or transposons libraries.

**Table 2:** Bacterial genes involved in attachment of pathogens on plants

Gene	Function	Pathogen	Plant/part	Method	Maximal effect of mutant	Reference
*adrA*	Regulation of cellulose biosynthesis	*S.* Newport and *S*. Enteritidis	Alfalfa sprouts	Directed deletion	expressed on the sprouts, but no effect in attachment	(Barak *et al*., 2007)
*bcsA*	Cellulose biosynthesis	*S*. Enteritidis	Alfalfa sprouts	Directed deletion	1 log reduction in attachment	(Barak *et al*., 2007)
*bcsC*	Cellulose biosynthesis	*S.* Typhimurium	Tomato fruits	Directed deletion	1 log reduction in attachment	(Shaw *et al*., 2011)
*csgB/csgD*	Curli subunit/regulatory protein	*S.* Newport	Alfalfa sprouts	Transposon insertion	Eightfold reduction in attachment	(Barak *et al*., 2005)
*csgA (agfA)*	Curli subunit	*E. coli* O157:H7	Alfalfa sprouts	Directed deletion	Fourfold reduction in attachment	(Torres *et al*., 2005)
*csgA (agfA)*	Curli subunit	*E. coli* K-12 and *E. coli* O157:H7	Lettuce leaves	Directed deletion	1 log reduction in attachment in the first 30 min	(Fink *et al*., 2012)
*csgA/bcsA*	Curli subunit/Cellulose biosynthesis	*S.* Typhimurium	Parsley leaves	Transposon insertion	No effect on attachment	(Lapidot *et al*., 2006)
*csgB/bcsA*	Curli subunit/Cellulose biosynthesis	*S*. Enteritidis	Alfalfa sprouts	Directed deletion	1.5 log reduction in attachment	(Barak *et al*., 2007)
*csgD (agfD)*	Regulatory protein	*E. coli* O157:H7	Alfalfa sprouts	Directed deletion	12-fold increase in attachment	(Torres *et al*., 2005)
*fliC*	Flagella	*E. coli* O157:H7	Spinach and lettuce leaves	Directed deletion	reduction in attachment	(Xicohtencatl-Cortes *et al*., 2009)
*fliC*	Flagella	*S.* Typhimurium and Senftenberg	Basil leaves	Directed deletion	Fivefold reduction in attachment in Senftenberg but not in Typhimurium	(Berger *et al*., 2009)
*fliC*	Flagella	*S.* Senftenberg	Tomato fruits	Directed deletion	No effect on attachment	(Shaw *et al*., 2011)
*fliC/fliB*	Flagella	*S.* Typhimurium	Tomato fruits	Directed deletion	No effect on attachment	(Shaw *et al*., 2011)
*escN*	T3SS	*E. coli* O157:H7	Arugula leaves	Directed deletion	No attachment	(Shaw *et al*., 2008)
*espB*	T3SS	*E. coli* O157:H7	Arugula leaves	Directed deletion	Twofold reduction in attachment	(Shaw *et al*., 2008)
*ompA*	Membrane protein	*E. coli* O157:H7	Alfalfa sprouts	Directed deletion	31-fold reduction in attachment	(Torres *et al*., 2005)
*pgaC*	poly-β-1,6-*N*-acetylglucosamine production	*E. coli* O157:H7	Alfalfa sprouts	Transposon insertion	3.7 log reduction in attachment	(Matthysse *et al*., 2008)
*rpoS*	Stationary-phase Sigma factor	*S.* Newport	Alfalfa sprouts	Transposon insertion	Eightfold reduction in attachment	(Barak *et al*., 2005)
*waaI*	LPS production	*E. coli* O157:H7	Alfalfa sprouts	Transposon insertion	1 log increment in attachment	(Matthysse *et al*., 2008)
*wcaD*	Colanic acid production	*E. coli* O157:H7	Alfalfa sprouts	Transposon insertion	2.9 log reduction in attachment	(Matthysse *et al*., 2008)
*wcaJ*	Colanic acid production	*S*. Enteritidis	Alfalfa sprouts	Directed deletion	No effect	(Barak *et al*., 2007)
*ycfR*	Putative membrane protein involved in biofilm	*E. coli* K-12 and *E. coli* O157:H7	Lettuce leaves	Transposon insertion	No effect	(Fink *et al*., 2012)
*ycfR*	Putative membrane protein involved in biofilm	*S.* Typhimurium and Saintpaul	Spinach leaves Grape tomatoes	Directed deletion	4 log reduction in attachment	(Salazar *et al*., 2013)
*yhjN*	Cellulose production	*E. coli* O157:H7	Alfalfa sprouts	Transposon insertion	1.8 log reduction in attachment	(Matthysse *et al*., 2008)
*yidE*	Adherence mediator	*E. coli* O157:H7	Alfalfa sprouts	Directed deletion	Fivefold increase in attachment	(Torres *et al*., 2005)
*yigG*	Putative inner membrane protein	*S.* Typhimurium and Saintpaul	Spinach leaves Grape tomatoes	Directed deletion	2 log reduction in attachment	(Salazar *et al*., 2013)
*yihO*	O-antigen capsule assembly	*S*. Enteritidis	Alfalfa sprouts	Directed deletion	1 log reduction in attachment	(Barak *et al*., 2007)

Strains of *E. coli* and *S. enterica* produce a diversity of pili and fimbriae and non-fimbrial adhesins that function as ‘professional’ adhesion systems as well as surface structures such as type III secretion systems (T3SSs) and flagella with alternative major functions (Hernandez-Reyes and Schikora, 2013; Yaron, 2014). Pili and fimbriae are hair-like appendages on the surface of the bacterial cells that often contain adhesins on their tips with affinity to different carbohydrates. Examples are the type 1, P, S and F1C fimbriae in *E. coli*. The adhesins interact with mammals' components, either non-specifically via hydrophobic or electrostatic interactions, or by binding to specific host cell receptor moieties, and are responsible to the tropism in adhesion to a specific host or tissue (Wagner and Hensel, 2011). Several adhesins and fimbriae of *E. coli* and *Salmonella* like amyloid curli fimbriae have widely been investigated in relation to adhesion to plants (Table 2). The studies demonstrated that curli usually have a role in attachment of *E. coli* and *Salmonella* to sprouts and leaves, but the effect of their inactivation is low. For example, deletion of *csg* genes resulted in no more than 1-log reduction in binding (Table 2). Furthermore, these results point out the complexity of adhesion and show that very little is known about the role of these adhesins in adherence of human pathogens to plant tissue. For example, mutations in the *csgA* gene had a very low effect on the ability of *E. coli* O157:H7 to bind to sprouts, but increased the binding of the same strain to Caco-2 human cells. On the other hand, insertion of *csgA* into the *E. coli* K-12 laboratory strain enabled the bacteria to bind to sprouts, indicating that *E. coli* O157:H7 possesses several redundant protein adhesins and that overexpression of each adhesin alone is sufficient to promote binding to alfalfa sprouts (Torres *et al*., 2005).

Taking adhesins of plant-associated bacteria into consideration indicates that additional factors that have hardly been investigated may have a role in attachment of enteric pathogens to plants. The type 1 fimbriae are widespread among members of the *Enterobacteriaceae* and are specified by their binding to mannosides in glycoproteins on the surface of mammalian cells. Type 1 fimbriae were also suggested to mediate adhesion of nitrogen fixating *Klebsiella* as well as *Klebsiella pneumoniae* to specific sites on the root hairs of bluegrass (Haahtela *et al*., 1985). Moreover, *E. coli* type V-secreted adhesins, such as the antigen 43, bind to integrins in the animal tissue via conserved RGD motifs (Henderson and Owen, 1999). Interestingly, plant pathogens also secrete RGD-containing proteins which might bind to specific receptor proteins of plants, but their involvement in adhesion has not been examined (Senchou *et al*., 2004).

Contradictory results have been obtained concerning the role of flagella in adherence of *Salmonella* and *E. coli* to plants. Deletion of the flagella subunit encoding gene *fliC* of *E. coli* O157:H7 or *S*. Senftenberg rendered the bacteria significantly less adherent to baby spinach and lettuce leaves (Xicohtencatl-Cortes *et al*., 2009), and leaf epidermis of basil (Berger *et al*., 2009), respectively, but deletion of the same gene in *S.* Typhimurium did not affect leaf attachment (Berger *et al*., 2009). It was suggested that this contradiction relates to the fact that the alternative flagellar subunit protein FliB is functional in this serovar, when FliC is not expressed. However, flagella were also not involved in attachment of *S*. Typhimurium to tomato fruits, even when both genes, *fliB* and *fliC*, were deleted (Shaw *et al*., 2011). In a further study, two genes of unknown function essential for swarming were found to be important factors for infection of alfalfa sprouts (Barak *et al*., 2009).

The role of biofilm formation in survival on the plant will be further discussed, but several genes products involved in production of biofilm components like *bcs* and *rpoS* have also a significant role in the initial attachment of *E. coli* and *Salmonella*. As can be seen in Table 2, the genes that have the most significant effect on the levels of attachment are genes involved in production of extracellular carbohydrates on the bacterial surface such as *pgaC* [synthesis of poly-β-1,6-*N*-acetylglucosamine (PGA)], *wcaD* (synthesis of colanic acid) in *E. coli* O157:H7 and *ycfR* (putative membrane protein involved in biofilm formation) in *Salmonella*. Mutants deficient in cellulose production reduced the ability of *E. coli* O157:H7 to attach to alfalfa sprouts (Matthysse *et al*., 2008), or the ability of *S*. Typhimurium to attach to tomato fruits (Shaw *et al*., 2011), but the influence of these mutations on the ability of the mutants to attach to other plants was much less noteworthy, with up to 1-log reduction. On the other hand, a plasmid carrying the cellulose gene allowed *E. coli* K-12 to bind to sprouts (Matthysse *et al*., 2008). Interestingly, the impact of each of these polysaccharides in attachment to mammalian cells and sprouts was different (Matthysse *et al*., 2008).

Collectively, these studies demonstrate that enteric pathogens specifically attach rapidly and irreversibly to produce surfaces. Attachment depends on plant and bacterial factors as well as on environmental conditions, but no single factor was found to be essential for attachment, possibly because bacteria use several parallel mechanisms to ensure tight attachment to different plants or to different plant cells under a wide variety of conditions. Moreover, despite the high numbers of publications in this topic, the exact contribution of each identified factor is not clear yet, probably due to redundancy in adhesion factors, diversity of adhesion factors in each pathogen and plant receptors, as well as the differences in cell surface composition.

## Biofilm of *S. enterica* and *E. coli* and its regulation

After or in parallel to attachment, the bacteria start to produce the biofilm matrix. The extracellular matrix produced by many *Enterobacteriaceae* is composed of proteinaceous components and exopolysaccharides. A major protein component is curli (amyloid fimbriae also known as thin aggregative fibres), which are encoded by at least seven genes organized in the *csgBAC* and *csgDEFG* operons (also termed *agf* genes). The *csgBA* genes encode the curli structural genes (Hammar *et al*., 1996), and the *csgDEFG* operon encodes, besides the major transcriptional regulator, CsgD, required for curli expression and biofilm formation, three accessory proteins required for the assembly of curli on the cell surface (Hammar *et al*., 1996; Loferer *et al*., 1997; Robinson *et al*., 2006). In *Salmonella*, the secreted large surface protein BapA (biofilm-associated protein) is also a major component of the biofilm matrix (Barnhart and Chapman, 2006; Latasa *et al*., 2006). Like fimbriae, this protein mediates the interactions between different cells leading to aggregation (Latasa *et al*., 2006).

A major exopolysaccharide in *S. enterica* and *E. coli* biofilms is cellulose (Zogaj *et al*., 2001), while some *E. coli* and *S. enterica* serovars also secrete capsular polysaccharides or other exopolysaccharides like colanic acid (Gibson *et al*., 2006). Many *E. coli* strains also have the potential to secrete PGA (Itoh *et al*., 2008). Cellulose consists of linear chains of glucose monomers connected by *β*-1,4-glycosidic bonds, which assemble into macromolecular fibrillar structures. These crystalline fibres are water insoluble and have a rigid structure (Ross *et al*., 1991). The two operons, *bcsABZC* and *bcsEFG*, encode the structural genes required for cellulose biosynthesis (Nobles *et al*., 2001; Zogaj *et al*., 2001; Solano *et al*., 2002), whereby BcsA and BcsB form the cellulose synthase complex (Romling, 2002; Omadjela *et al*., 2013).

*Escherichia coli* and *Salmonella* strains produce more than 80 distinct capsules which can also be a constituent of the biofilm matrix, and are classified into several groups. Group 4 capsules comprise of O-polysaccharides, structurally similar to the O-polysaccharides of the LPS, termed the O-antigen capsules. In *E. coli*, they are polymerized by the Wxy polymerase, and transferred across the membrane by the Wzx complex (Whitfield and Roberts, 1999). In *Salmonella*, two *yih* operons were found to be important for capsule assembly and translocation (Gibson *et al*., 2006).

**Fig 1:**
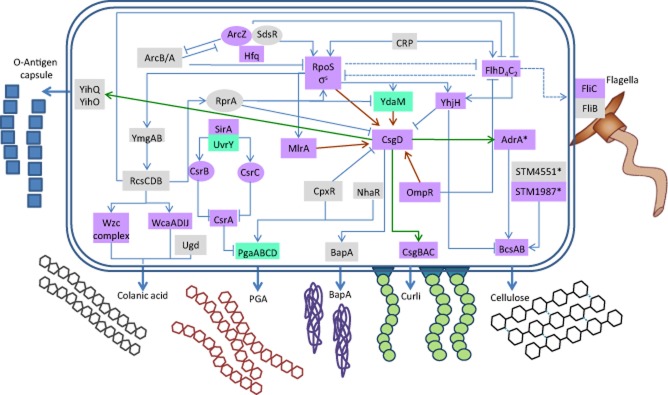
Regulation of components of the biofilm matrix in *Salmonella typhimurium* and *Escherichia* *coli.*Proteins and sRNAs controlling the synthesis of biofilm components are shown. Straight arrows: direct activation. Straight lines with blunt ends: direct inhibition. Dotted lines: indirect effects. Proteins are in boxes (green, *E. coli* only; grey, *S*. Typhimurium only; violet, in both species), and regulatory sRNAs are in circle. Cyclic di-GMP binding proteins are marked with an asterisk. Additional regulatory elements were not included for clarity. The figure is mainly based on data from the following references: (Gibson *et al*., 2006; Mika and Hengge, 2013; Anwar *et al*., 2014).

A regulatory scheme for the main biofilm components is illustrated in Fig. 1. For clarity, only major regulators and regulators that have been investigated in relation to association of the bacteria with plants are shown. As outlined above, the extracellular matrix components, cellulose, curli fimbriae and in *S. enterica* BapA are positively regulated by CsgD, the major hub of biofilm formation (Romling *et al*., 2000; Uhlich *et al*., 2001; Latasa *et al*., 2006; Simm *et al*., 2014). Synthesis of the *Salmonella* O-antigen capsule coregulates with the cellulose synthesis. CsgD also regulates the *yih* genes in coordination with cellulose and curli (Gibson *et al*., 2006). Regulation of CsgD and subsequently the expression of the matrix components is highly responsive to many environmental signals such as growth phase, nutrients, oxygen tension, ethanol, temperature, osmolarity and a number of regulatory proteins (Gerstel and Romling, 2003). For most strains of *Salmonella* and also a fraction of *E. coli* strains, *csgD* expression is optimal at temperatures below 30°C in media with low salt (Romling *et al*., 1998; Bokranz *et al*., 2005). Maximal expression is observed during stationary phase upon limitation of nutrients such as nitrogen, phosphate and iron (Gerstel and Romling, 2003), which, directly and indirectly, requires the stationary-phase sigma factor RpoS (Arnqvist *et al*., 1994). The response regulator OmpR, a component of the two-component regulatory system OmpR/EnvZ that responds to changes in osmolarity (Pratt *et al*., 1996), is absolutely required for CsgD expression. Oxygen tension also plays a major determinative role in CsgD expression (Romling *et al*., 1998; Gerstel and Romling, 2001). Investigating 51 *S.* Typhimurium strains from different origins indeed demonstrated that most strains form optimal biofilm at acidic pH (∼ 5.5), 0.5% NaCl and 25°C (Lianou and Koutsoumanis, 2012). In the same line, the genes involved in curli and cellulose production were highly induced in a panel of *S. enterica* strains at 25°C and low nutrient availability (Castelijn *et al*., 2012), and also colanic acid is generally not produced at temperatures above 30°C (Whitfield and Roberts, 1999). PGA is synthesized at 37°C, but it is also implicated in attachment to surfaces during growth at lower temperatures (Wang *et al*., 2004).

Regulation of cellulose biosynthesis by CsgD is indirect. Once CsgD is expressed in the stationary phase of growth, it activates the transcription of *adrA* encoding a diguanylate cyclase that subsequently leads to cellulose biosynthesis through the production of the allosteric activator cyclic di-GMP (Romling *et al*., 1998; 2000). However, cellulose biosynthesis can be uncoupled from CsgD expression, as alternative diguanylate cyclases encoded by the *S. enterica* chromosome like STM4551 and STM1987 can activate cellulose biosynthesis under alternative growth conditions (Anwar *et al*., 2014; Simm *et al*., 2014). Cyclic di-GMP has emerged as a major regulatory secondary messenger signalling system activating *csgD* expression in *S. enterica* and *E. coli* (Römling *et al*., 2013), and is usually with oppositely regulated with the flagella synthesis and function (Mika and Hengge, 2013). Regulation of csgD expression by cyclic di-GMP is complex and involves at least four di-guanylate cyclases and four phosphodiesterases in *S.* Typhimurium.

Hfq, a RNA chaperone, is another central positive regulator of biofilm formation in *S*. Typhimurium and *E. coli* (Holmqvist *et al*., 2010; Monteiro *et al*., 2012). In *S*. Typhimurium, Hfq together with its associated small RNAs ArcZ and SdsR positively control the expression of CsgD. ArcZ also regulates the transition between sessility and motility, and the timing of type 1 fimbriae versus curli fimbriae surface attachment at ambient temperatures (Monteiro *et al*., 2012).

Recently, it has been shown that hyper-expression of the T3SS-1 of *Salmonella* facilitates a cell aggregation phenotype that results in biofilm formation (Jennings *et al*., 2012). A similar observation was also described in the plant pathogen *Erwinia chrysanthemi* (Yap *et al*., 2005). This biofilm is not dependent on cellulose, curli or flagella production, and its extracellular matrix contains different virulence proteins secreted by the T3SS-1 system like SipA and SopB (Jennings *et al*., 2012). Other types of biofilms of *S.* Typhimurium are mainly dependent on type 1 fimbriae (Monteiro *et al*., 2012) and flagella (Crawford *et al*., 2010).

## Biofilm formation by *S. enterica* and *E. coli* on produce surfaces and its role in survival on plants

The formation of biofilms by plant epiphytic or pathogenic bacteria has long been known (Danhorn and Fuqua, 2007); however, the discovery that human enteric pathogens are able to establish biofilms on plant surfaces was unexpected. *Escherichia coli*, *Salmonella*, *Campylobacter*, *Listeria* and *Shigella* have been found to form distinct biofilms on surfaces of produce such as tomatoes, melons and parsley (Agle, 2003; Annous *et al*., 2005; Iturriaga *et al*., 2007). Consequently, the number of studies on the formation of biofilms by foodborne pathogens on produce surfaces has expanded in the last decade only. A correlation between the ability to form biofilms and the attachment to fresh produce and/or survival was shown as strains with pronounced biofilm formation *in vitro* also attached to plants in significantly higher numbers, or survived better after disinfection (Lapidot *et al*., 2006; Patel and Sharma, 2010). This correlation extended to strains isolated from produce-related outbreaks. Isolates of *Salmonella* collected during tomato outbreaks produced biofilms and better adhered and attached to tomato leaflets compared with non-biofilm-producing strains (Cevallos-Cevallos *et al*., 2012). The *E. coli* O104:H4 strain that caused the devastating fenugreek seed outbreak in 2011 possesses a unique composition of multiple virulence factors, as well as the ability to produce rapidly a more stable and thicker biofilm compared with *E. coli* O157:H7. The ability of this strain to form a biofilm is probably aided by the production of high levels of exopolysaccharides and fimbriae due to overexpression of *pgaA* and *aggR* genes products, involved in the production of PGA, and regulation of type 1 aggregative adherence fimbriae respectively (Al Safadi *et al*., 2012). Also, *E. coli* strains isolated from plants formed significantly more biofilms and produced more cellulose and curli compared with *E. coli* strains isolated from human and other animals (Meric *et al*., 2013). Similarly, *Salmonella* isolates from produce commodities formed significantly thicker biofilms and persisted on spinach plants at higher numbers than poultry *Salmonella* isolates. Increasing persistence of the isolates was attributed to curli expression in several isolates. However, *S*. Tennessee formed a greater biofilm, but produced less curli, suggesting that other extracellular appendages contribute to attachment and biofilm formation (Patel *et al*., 2013). In contrast to the observations indicating that the ability to produce a biofilm improves the survival of the pathogens on the plant, recent evidence has shown that during adaptation of *S.* Typhimurium in green tomato fruits, the bacteria lose the ability to produce biofilms (Salazar *et al*., 2013). In line with this finding, a survey of the *Salmonella* strains recovered from produce-associated outbreaks revealed that many of them do not synthesize cellulose and/or curli (Zaragoza *et al*., 2012). Similarly, the majority of *E. coli* O157:H7 strains isolated from various outbreaks do not express curli and show only weak or non-biofilm formation *in vivo* (Liu *et al*., 2014). The reasons for these opposing observations are not clear.

Bacteria embedded within biofilms on plant tissue are more difficult to remove and more resistant to inactivation than their planktonic counterparts (Chmielewski and Frank, 2003). Biofilm-associated cells as well as capsule producing cells are also significantly more tolerant to desiccation, as deletion of CsgD, the major biofilm activator in *Salmonella*, resulted in increased susceptibility to desiccation stress (Gibson *et al*., 2006). Generally, in recent years, the role of biofilm-related genes in survival on sprouts, leaves or fruits of different plants was investigated (Table 3). The most investigated genes demonstrated various effects under different experimental conditions. For example, deletion of the cellulose synthase gene *bcsA* in *Salmonella* serovars resulted in a significant reduction in survival on alfalfa sprouts (Barak *et al*., 2007), but caused no effect on tomatoes (Noel *et al*., 2010). The genes with the most significant effect in survival on both alfalfa sprouts and tomatoes were the *yih* genes, involved in the synthesis of O-antigen capsule in *Salmonella* (Table 3).

**Table 3:** Bacterial genes involved in biofilm formation and/or survival on plants

Gene	Function	Pathogen	Plant/part	Method	Comments	Reference
*adrA*	Regulation of cellulose synthesis	*S.* Newport	Alfalfa sprouts	Directed deletion	No effect on survival 24–48 hpi	(Barak *et al*., 2007)
*aroA*	5-enolpyruvylshikimate 3-phosphate synthase	*S.* Typhimurium	Red tomatoes	Directed deletion	reduction in survival	(Noel *et al*., 2010)
*bcsA*	Cellulose synthase	*S*. Enteritidis	Alfalfa sprouts	Directed deletion	2 log reduction in survival	(Barak *et al*., 2007)
*bcsA*	Cellulose synthase	*S.* Typhimurium	Red tomatoes	Directed deletion	No effect on survival	(Noel *et al*., 2010)
*csgB/bcsA*	Curli/Cellulose	*S*. Enteritidis	Alfalfa sprouts	Directed deletion	1.5 log reduction in survival	(Barak *et al*., 2007)
*csgA*	Curli subunit	*E. coli* K-12 and *E. coli* O157:H7	lettuce leaves	Transposon insertion	0.5–3 log reduction in survival	(Fink *et al*., 2012)
*csgA*	Curli subunit	*E. coli* O157:H7	Spinach grown on hydroponics and in soil	Directed deletion and overexpression	No effect on root uptake and internalization	(Macarisin *et al*., 2014)
*csgB/csgC*	Curli subunit and curli assembly	*S.* Typhimurium	Red tomatoes	Directed deletion	No effect on survival	(Noel *et al*., 2010)
*csgD*	Regulation of biofilm and *rdar* morphotype	*S.* Typhimurium	Tomato leaves and fruits	Directed deletion and point mutation	*rdar* morphotype better survived in the plant	(Gu *et al*., 2011)
*csrB/csrC*	sRNAs, Global regulation	*S.* Typhimurium	Red tomatoes	Directed deletion	No effect on survival	(Noel *et al*., 2010)
*cysB*	Regulation of cysteine operon and antibiotic resistance	*S.* Typhimurium	Red tomatoes	Directed deletion	reduction in survival	(Noel *et al*., 2010)
*flhDC*	Flagella regulators	*S.* Typhimurium	Red tomatoes	Transposon insertion	No effect on survival	(Noel *et al*., 2010)
*flhF*	Flagella	*S.* Typhimurium	Red tomatoes	Directed deletion	No effect on survival	(Noel *et al*., 2010)
*hilA*	Regulator of virulence genes	*S.* Typhimurium	Red tomatoes	Directed deletion	Trend of improved survival	(Noel *et al*., 2010)
*lpfA*	Long polar fimbriae	*S.* Typhimurium	Red tomatoes	Directed deletion	No effect on survival	(Noel *et al*., 2010)
*motA*	Flagella motor	*S.* Typhimurium	Red tomatoes	Transposon insertion	Improved survival	(Noel *et al*., 2010)
*pgaC*	Poly-β-1,6-*N*-acetylglucosamine production	*E. coli*	Alfalfa sprouts	Transposon insertion	Decreased biofilm formation	(Matthysse *et al*., 2008)
*sirA*	Type VI secretion protein	*S.* Typhimurium	Red tomatoes	Directed deletion	Improved survival	(Noel *et al*., 2010)
*sirA*	Type VI secretion protein	*S.* Typhimurium and Saintpaul	Spinach leaves Grape tomatoes	Directed deletion	2 log reduction in survival	(Salazar *et al*., 2013)
*wcaD*	Colanic acid production	*E. coli*	Alfalfa sprouts	Transposon insertion	Lowers biofilm formation	(Matthysse *et al*., 2008)
*wcaJ*	Colanic acid production	*S*. Enteritidis	Alfalfa sprouts	Directed deletion	No effect on survival	(Barak *et al*., 2007)
*ycfR*	Putative membrane protein involved in stress response and biofilm	*E. coli* K-12 and *E. coli* O157:H7	Lettuce leaves	Transposon insertion	1.5–3 log reduction in survival	(Fink *et al*., 2012)
*yhjN*	Cellulose production	*E. coli*	Alfalfa sprouts	Transposon insertion	Lowers biofilm formation	(Matthysse *et al*., 2008)
*yihO*	O-antigen capsule assembly	*S*. Enteritidis	Alfalfa sprouts	Directed deletion	2.5 log reduction in survival 24 hpi	(Barak *et al*., 2007)
*yihT*	O-antigen capsule assembly	*S.* Typhimurium	Green and red tomatoes	Directed deletion	2–3 log reduction in survival 34 dpa on green tomatoes and in fruits defective in ethylene perception	(Marvasi *et al*., 2013)

Presumably, these and other mechanisms aid long-term survival of biofilm-associated cells on the plant surface not only in the field but also during harvest, transport, sanitation and storage. On parsley plants, for instance, resistance to disinfection treatments (i.e. chlorination) was improved in biofilm producing *S.* Typhimurium compared with non-producing mutants, when washing was conducted a week after inoculation, but no differences were observed when the treatment was carried out a few hours after inoculation (Lapidot *et al*., 2006).

## Regulation of biofilm formation on plants

As discussed above, several environmental conditions were shown to have an impact on biofilm production. For example, biofilm formation of *Salmonella* has been reported to be maximal under reduced nutrient availability, aerobic conditions, low osmolarity and mid temperatures (25–28°C) (Gerstel and Romling, 2003). All these conditions exist on a plant surface rather than in the gut environment. Indeed, several studies reported about induction of biofilm-associated genes in the plant environment. Assessment of the transcriptional profile in *E. coli* O157:H7 attached to intact lettuce leaves showed that 10% of the genes have at least a twofold expression change between day 0 and day 1 and/or day 3 (Fink *et al*., 2012). Among them, *csgA* and *csgB* showed 34.6-fold and 13.9-fold induction, respectively, and *rpoS* 2.1-fold induction. Other genes probably involve in biofilm regulation, such as *ybiM* and *yceP*, also show a very strong induction (Fink *et al*., 2012). In line with these findings, *E. coli* attached to the lettuce rhizosphere upregulates the *csg* genes (Hou *et al*., 2012), and *S.* Weltevreden upregulated *csg* and TTSS-2 genes during alfalfa sprout colonization as compared with M9 minimal medium (Brankatschk *et al*., 2013). Screening for *Salmonella* genes differentially regulated in tomatoes relative to growth in Luria–Bertani soft agar identified several genes, including genes associated with attachment, stress response, biofilm formation and capsule formation (Noel *et al*., 2010). Furthermore, deletion of SirA, involved in regulation of type 1 fimbriae and biofilm formation (Teplitski *et al*., 2006), and MotA, involved in motility, modestly affected fitness, while YihT, involved in synthesis of the capsule, affected the fitness in green, but not ripe tomatoes. In these studies, known *Salmonella* genes associated with regulation of motility and virulence in animals, such as *hilA*, *flhDC* and *fliF*, did not contribute to the fitness of the bacteria (Noel *et al*., 2010; Marvasi *et al*., 2013).

## Incorporation of pathogens in multi-species biofilms

Bacterial cells introduced to the leaf surface, if not producing their own aggregates or biofilms, have a better chance of surviving when they are deposited on or in aggregates of existing bacteria (Monier and Lindow, 2005). *Salmonella enterica* serovar Thompson and the plant pathogen *Pantoea agglomerans*, for instance, were shown to form co-aggregates on cilantro leaves (Brandl and Mandrell, 2002), and the epiphytes *Wausteria paucula* supported the survival of *E. coli* O157:H7 on lettuce leaves (Cooley *et al*., 2006). Also, the fungal plant pathogens *Cladosporium cladosporioides* and *Penicillium expansum* promoted the colonization of *Salmonella* in cantaloupe (Richards and Beuchat, 2005). A recent study has shown that *E. coli* O157:H7 strains promote biofilm formation of some strong biofilm-producing species isolated from fresh produce processing facilities, such as *Burkholderia caryophylli* and *Ralstonia insidiosa*. Likewise, the population of *E. coli* increases by 1 log in the dual-species biofilms (Liu *et al*., 2014). These studies demonstrate that indigenous microorganisms may aid attachment and long-term survival of foodborne pathogens on the plant surface and during washing with antimicrobials. However, it is not clear whether this positive effect results from embedding of foodborne pathogens in the already existing biofilms or from other interactions like lesion and release of nutrients.

## The plant response and its effect on bacterial biofilms

Discussion of plant-associated biofilms is not completed without referring to the plant defence response. Plants apply a range of mechanisms for protection against microorganisms. The plant protection includes local or systemic production of defence enzymes and antimicrobial molecules. Some mechanisms, like the production of essential oils with a broad-range antimicrobial activity, are constitutively active, while others, like secretion of reactive oxygen species (ROS), are induced after exposure to pathogens. Evidence indicates that plants apply similar mechanisms of defence in response to internalization of human enteric pathogens as against plant pathogens. Microorganisms encounter surface receptors on host cells, termed pattern recognition receptors (PRRs), that recognize conserved pathogen-associated molecular patterns (PAMPs) and trigger downstream defence signalling pathways. PAMPs include conserved microbial surface molecules such as flagellin, LPS and glycoproteins (Nurnberger *et al*., 2004). Recognition of PAMPs by PRRs initiates PAMP-triggered immunity (PTI), which usually prevents microbial growth and halts infection before the microorganism gains a hold in the plant (Chisholm *et al*., 2006). Many surface components of *E. coli* and *Salmonella* such as LPS, flagella, peptidoglycans, curli and pili are homologous to PAMPs of plant pathogens. Deletion of these PAMPs from *E. coli* or *Salmonella* usually resulted in better colonization of the interior of the plants than the wild-type strains (Iniguez *et al*., 2005; Seo and Matthews, 2012). Some of these molecules are also components of the biofilm matrix. Curli can serve as a PAMP (Seo and Matthews, 2012), and the *S*. Typhimurium LPS being a strong PAMP in tobacco plants (Shirron and Yaron, 2011). The flagellin subunit FliC, but not FliB, is recognized by *Nicotiana benthamiana* and tomato plants as a PAMP and activates their PTI (Meng *et al*., 2013). These observations indicate that components of the bacteria and the biofilm matrix may induce the plant response. On the other hand, biofilm production may mask the underlying bacterial surface, providing further protection. Indeed, an *E. coli* O157:H7 mutant that produces a great amount of exopolysaccharides and a thick capsule exhibits a better survival pattern on *Arabidopsis* compared with the wild-type strain (Meng *et al*., 2013).

The response of the plant cells results in production of ROS, nitric oxide, different ions as well as molecular signals like jasmonate, salicylic acid and ethylene (Jones and Dangl, 2006). Some of these compounds were found to have a role not only against persistence of enteric pathogens in plants but also against biofilm formation. Ethylene and salicylic acid decreased endophytic colonization of *Salmonella* in alfalfa (Iniguez *et al*., 2005). Interestingly, an inverse correlation was observed between *in vitro* biofilm formation by uropathogenic *E. coli* and the salicylate concentration (Vila and Soto, 2012). Moreover, the synthesis of fimbriae such as P fimbriae and type 1 fimbriae was reduced following growth in the presence of salicylate (Kunin *et al*., 1994). In the case of type 1 fimbriae, salicylate decreased the expression of *fimA* coding for its major structural subunit (Vila and Soto, 2012). Similarly, jasmonic acid reduced the synthesis of the O-antigen capsule by *Salmonella* in tomatoes. In mammals, the *yih* operon of *Salmonella* is induced by bile and enhances biofilm formation on gallbladder by triggering the O-antigen capsule production. In tomatoes, on the other hand, jasmonic acid and its precursors, produced during the plant response, strongly reduce the expression of YihT, and thus inhibit biofilm formation (Marvasi *et al*., 2013).

Other microorganisms in the plant environment can also affect, directly or indirectly, through induction of the plant defence response, the biofilm formation by human enteric pathogens. The soil bacterium *B. subtilis* colonizes roots of different plants and is currently used as a biocontrol agent against plant pathogens. During colonization of the roots, it produces surfactin, an antimicrobial lipopeptide that not only inhibits the growth of common plant pathogens, but also induces the plant defence response (reviewed in Vlamakis *et al*., 2013). Interestingly, surfactin molecules also delay adhesion of *Salmonella* and *Listeria* strains to solid surfaces (Nitschke *et al*., 2009), and inhibit biofilm formation by *Salmonella* (Mireles *et al*., 2001). However, their influence on colonization of enteric pathogens on plants has not been investigated yet.

In addition to the plant immune response triggered upon exposure to microorganisms, many plants produce antimicrobial compounds constitutively. Interestingly, some of these compounds do not only inhibit bacterial growth, but also inhibit biofilm formation or even efficiently kill the pathogens specifically in biofilms. For example, 3% of 498 investigated plant extracts inhibited biofilm formation of *E. coli* O157:H7. The most active extract, *Carex dimorpholepis*, inhibits curli formation and decreases swimming and swarming motility, probably by repression of quorum sensing genes (Lee *et al*., 2013). In another study, *Carex* plant extracts inhibited *E. coli* O157:H7 and *Pseudomonas aeruginosa* biofilm formation without affecting planktonic cells growth. One of the active antibiofilm compounds in these extracts was ε-viniferin (Cho *et al*., 2013). Plant sterols such as the β-sitosterol glucoside from citrus are potent inhibitors of *E. coli* O157:H7 biofilm formation and motility, without affecting the cell viability. This inhibition probably occurs through repression of *rssAB* and *hns*, regulatory elements of the flagellar operon (Vikram *et al*., 2013). Essential oils from cassia (*Cinnamomum aromaticum*) and Peru balsam (*Myroxylon balsamum*) kill plant and human pathogens within biofilms in similar efficacy as the planktonic cells, and the oil of red thyme (*Thymus vulgaris*) is even more effective against biofilms cells than planktonic bacteria (Kavanaugh and Ribbeck, 2012). *Helichrysum italicum* produces several metabolites such as acylated styrylpyrones derivatives with anti-biofilm properties against *P. aeruginosa* (D'Abrosca *et al*., 2013).

In conclusion, regulation of biofilm formation on the plant is a complex process affected by many factors. There are several levels of interactions of the plant response with bacteria and their biofilms. Current study indicates a role of biofilm formation in triggering or evading the plant response, while molecules produced by the plant either constituently or in response to the bacteria may trigger or inhibit the process of biofilm formation.

## Conclusion

**Fig 2:**
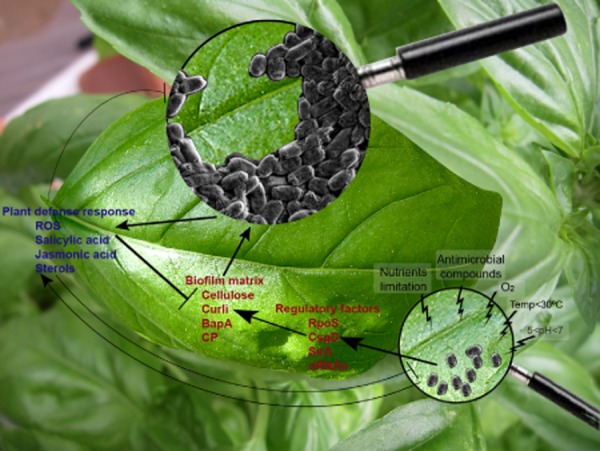
Illustration of biofilm formation by *Salmonella* cells on a leaf.Upon attachment of *Salmonella* cells to the leaf, the bacteria are exposed to environmental conditions (temperature below 30°C, atmospheric oxygen, etc.) that trigger expression of regulatory sRNAs and proteins such as RpoS, CsgD and SirA. Expression of these proteins and sRNAs is enhanced by stress signals existing on the leaf surface such as low availability of nutrients, and activity of antimicrobial compounds produced by the plant or indigenous microorganisms. The induced regulatory proteins activate the genes involved in production of components of the biofilm matrix such as cellulose, curli, BapA and capsules (CP), leading to the development of biofilms on the leaf surface. While the biofilm structure stabilizes the colonization on the plant and provides protection from different stresses, its components also contribute to the induction of the local and systemic plant defence response. As part of the plant response, triggered by both, single bacteria and biofilms, the plant produces and secretes different signal and antimicrobial compounds such as ROS compounds, salicylic acid, jasmonic acid and sterols. Some of these compounds kill free and biofilm associated bacteria and/or inhibit the process of biofilm formation.

Most likely, plants do not provide optimal conditions for growth of human enteric pathogens, but the pathogens use different mechanisms to tightly attach to favourable sites on the plant organs and to fit the harsh conditions in order to survive in the plants long enough and in sufficient numbers for successful infection of new mammalian hosts with the consequence of a significant number of outbreaks. The studies detailed in this review indicate that production of biofilms is a common strategy employed by pathogenic *Salmonella* and *E. coli* strains to survive in different niches of the plant. Following a tight adhesion on favourable sites, the process of biofilm formation on the plant is affected by many environmental factors, as well as properties of the plant and characters of the pathogenic strain or the ingenious plant microflora. The synthesis of the biofilm matrix components such as cellulose and curli occurs under environmental conditions similar to the ones existing on the plants and may be further induced or inhibited by compounds produced by the plants or neighbouring microorganisms (Fig. 2). Although intensive research activity is ongoing, our current understanding of the factors that affect biofilm formation and its role in survival of foodborne pathogens on the plants is rudimentary. The different strains, serovars and pathovars and a wide variety of host plants in combination with diverse experimental methods of inoculation and plant growth, limit the ability to compare the results of different studies and to unambiguously conclude the dominant factors for adhesion and biofilm formation.

Recent evidence has shown that several plant hosts and environmental plant-associated bacteria such as *B. subtilis* are able to synthesize and secrete compounds that not only target planktonic cells, but also inhibit attachment and biofilm formation or even specifically kill bacteria in biofilms. Future studies on plant-related compounds or plant symbionts that affect bacterial biofilm formation are required and might discover novel substances involved in biofilm formation. Subsequently, understanding of the mode of action of such compounds will aid in development of novel strategies to decrease the fitness of human enteric pathogens on fresh produce and prevent outbreaks, and also in inhibition of biofilm formation not only on such foodstuff, but also on many other types of solid surfaces of medical, industrial and agricultural importance.
